# Contrast response in a comprehensive network model of macaque V1

**DOI:** 10.1167/jov.20.4.16

**Published:** 2020-04-24

**Authors:** Logan Chariker, Robert Shapley, Lai-Sang Young

**Affiliations:** Center for Neural Science, New York University, New York, NY, USA; Courant Institute of Mathematical Sciences, New York University, New York, NY, USA

**Keywords:** visual cortex, computational model, contrast response, mechanisms

## Abstract

The response to contrast is one of the most important functions of the macaque primary visual cortex, V1, but up to now there has not been an adequate theory for it. To fill this gap in our understanding of cortical function, we built and analyzed a new large-scale, biologically constrained model of the input layer, 4Cα, of macaque V1. We called the new model CSY2. We challenged CSY2 with a three-parameter family of visual stimuli that varied in contrast, orientation, and spatial frequency. CSY2 accurately simulated experimental data and made many new predictions. It accounted for 1) the shapes of firing-rate-versus-contrast functions, 2) orientation and spatial frequency tuning versus contrast, and 3) the approximate contrast-invariance of cortical activity maps. Post-analysis revealed that the mechanisms that were needed to produce the successful simulations of contrast response included strong recurrent excitation and inhibition that find dynamic equilibria across the cortical surface, dynamic feedback between L6 and L4, and synaptic dynamics like inhibitory synaptic depression.

## Introduction

Our scientific aims are to use computational models to understand the neural mechanisms responsible for cortical function. This article is about contrast response in the macaque monkey's primary visual cortex, V1. We used a new large-scale model (called CSY2) to gain insight into mechanistic explanations for contrast response and cortical dynamics.

Arguably the most important function of the magnocellular pathway of the primary visual cortex, V1, is contrast response (e.g., Albrecht & Hamilton, 1982), yet so far there has not been an adequate theoretical account of how the firing rate of visual cortical cells depends on visual contrast. The mechanisms for contrast response may seem straightforward: at higher contrast, there is more spatial and temporal modulation of the rate at which photons hit the retina, which sends a stronger modulated signal to the lateral geniculate nucleus (LGN), which then passes it to the cortex, increasing the firing rate of cortical neurons. This line of reasoning assumes that the cortical contrast response is inherited from the retina. It assumes implicitly that cortical neurons function as filters, or transducers, that transform currents into spikes in a mostly feedforward regime. However, the modern view of cat and monkey cortex (e.g., Douglas & Martin, 2007) is that cortical signal transmission is much less feedforward than previously thought, even in the input layers. Data on the extreme sparsity of feedforward LGN input to macaque V1 (Connelly & Van Essen 1984; Silveira & Perry 1991) support the modern view and specifically require corticocortical interaction to provide most of the excitation to cortical neurons.

To reconcile experimental data with the modern recurrent view of cortical function, we need a new theory of contrast response in the cortex. An adequate theory must take into account that the LGN supplies no more than 10% to 20% of the excitatory synaptic input to cortical neurons; the bulk of cortical excitation comes from excitatory interactions within the cortex (Douglas & Martin, 1991, 2007). This leads immediately to the following three fundamental questions about contrast response in the cortex:

1) How exactly do cortical neurons respond with so many spikes in a graded manner following the changes in such a small feedforward input?
2) How do these responses together produce the increased feature selectivity of cortical neurons at high contrast?
3) If cortical response is dominated by corticocortical interactions, how do V1 neurons that are nearby in the cortical network but prefer different orientations respond so differently to increased contrast?

This article offers insight into these three questions based on analysis of a biologically realistic model. The issues are discussed in Results (1) to (3).

Previously, descriptive explanations were proposed for contrast response in terms of cortical contrast gain control (Ohzawa et al., 1982) or normalization (Carandini & Heeger, 2011) but these were not intended as mechanistic explanations. Previous mechanistic models did not deal with the extreme sparsity of LGN input (Somers et al., 1995; Tao et al., 2004; Troyer et al., 1998). We compare CSY2 with previous models in the Discussion.

Earlier we built a large-scale network model called CSY1, a model of layer 4Cα (L4) in the macaque visual cortex (Chariker et al., 2016). We chose to study L4 because orientation and spatial frequency (sf) selectivity are observable already in this cortical input layer (Livingstone & Hubel, 1984; Ringach et al., 2002). The CSY1 model simulated many aspects of V1 behavior, including orientation and sf selectivity and their population distributions, as well as average values. CSY1 was the first V1 model that included the extreme sparsity of LGN inputs. But CSY1 was designed to simulate only spontaneous activity and responses to stimuli at full contrast.

We present in this article a new model, CSY2, based on CSY1. The CSY2 model is capable of simulating cortical responses to the full range of contrast while retaining all the capabilities of CSY1. CSY2 is a mechanistic model composed of integrate-and-fire neurons that have excitatory and inhibitory synapses. The fundamental mechanisms that determine its contrast response are the recurrent excitatory and inhibitory synaptic interactions within the cortical network. Recurrent circuitry is a feature of cerebral cortex (Braitenberg & Schüz, 1998; Douglas & Martin, 1991, 2007; Thomson et al., 2002) that influences many functions of the cortex. To enable CSY2 to explain cortical contrast response, we had to incorporate into CSY2 some additional known cortical mechanisms. For instance, CSY2 has a dynamic feedback loop between layer 6 (L6) and layer 4 (Callaway, 1998). When presented with a visual stimulus, feedforward input from the LGN causes neurons in L4 and L6 in CSY2 to interact with one another dynamically to compute a response, mimicking the way neurons compute in the real cortex. As presented in the Results, the successful simulation of V1 visual responses across visual contrasts by CSY2 is a big step in being able to understand the function of V1 theoretically.

## Methods (model description)

This section contains a description of CSY2, the model presented in this article, as well as its predecessor CSY1, on which CSY2 was built.

### (1) Overview: CSY2, a model of LGN→L4↔L6

**Figure 1.**
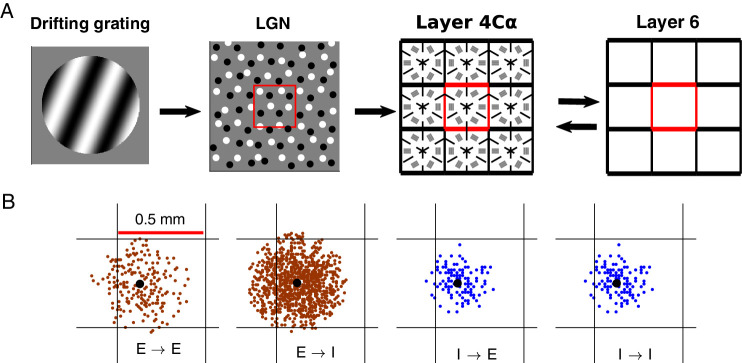
The CSY2 model at a glance. (A) Model flow diagram. The grating on the left is symbolic of the visual stimulus presented to the eye. It is presented in the form of a light intensity map I(*x*,*t*) where *x* denotes the location on the retina and *t* the time; I(*x*,*t*) can be any function. The CSY2 model begins with the modeling of LGN as ON/OFF cells responding to I(*x*,*t*), the red square at the center of the LGN sheet corresponding roughly to one hypercolumn (HC) in cortex. LGN projects to L4. The right half of the diagram depicts a feedback loop between L4 and L6. Our main focus is on L4: The model has nine HCs, each subdivided into six intended orientation domains; a majority of the neurons in each domain is connected to LGN configurations spatially aligned with the intended orientation [details in Chariker et al. (2016)]. (B) Neuronal connectivity within L4. Black dot is a postsynaptic neuron, and red or blue dots are distribution of its presynaptic cells, for E to E, E to I, and I to E/I, respectively.

The physical layout of CSY2 is as shown in Figure 1A. As in CSY1, the inputs to CSY2 are visual images of drifting grating patterns. The primary focus of CSY2 is the region of L4 that represents the visual field at about 5° eccentricity because we have many data about this region of V1 with which to compare model and data. Also modeled is the corresponding region of the thalamic LGN that projects to L4. Magnocellular LGN responses are modeled as spatiotemporal filters of the visual image (see Supplementary Appendix section 0.1). CSY2 also models the corresponding region of L6 of V1, which is known to feedback to L4 (Callaway, 1998). Signal transmission from LGN to L4 is assumed to be feedforward only, whereas the interaction between L4 and L6 is bidirectional as indicated in Figure 1. Inputs to the model are visual stimuli represented as time-dependent light-intensity maps I(*x*,*t*) where *x* denotes the location on the retina (or on the LGN sheet) and *t* denotes the time. Once I(*x*,*t*) is presented to the model, the LGN will compute a response, which is passed to L4, and the dynamic interaction between L4 and L6 produces a response. Outputs of the CSY2 model are shown in the Results. Only drifting gratings are used in this article but the model's capabilities are not limited to this class of visual stimuli.

CSY1 has a similar structure as that shown in Figure 1A, minus the bidirectional interaction between L4 and L6. Because it was built to respond only to gratings at full contrast, the contribution of L6 in CSY1 was inserted by hand. A self-adjusted feedback mechanism capable of responding, in principle, to any stimuli presented to the eye was introduced in CSY2. Another novel feature of CSY2 was the use of synaptic and adaptation dynamics, which were absent (because they are not needed) in CSY1.

Here we first review the construction and properties of CSY1 before proceeding to the new features of CSY2, with further technical information provided in the Supplementary Appendix.

### (2) Review of the previously constructed model CSY1

The material on CSY1 is taken mostly from (Chariker et al., 2016, 2018). We begin with a description of the model architecture.

#### Magnocellular LGN layers

An important new feature of CSY1—which is also the basis for our contention that a mechanistic explanation for contrast response is needed—is the very sparse LGN input to macaque primary visual cortex, V1. There are on average approximately 10 LGN cells (5 ON and 5 OFF) that correspond to each hypercolumn (HC). Because these numbers are important, we review the anatomic evidence and repeat the computation here.

Silveira and Perry (1991) found that the M retinal ganglion cell density at 5° eccentricity is 3500/mm^2^. If we take the conversion factor of {200 µm (on the retina)}/{degree visual angle} for the Java monkey *M* *fascicularis* (Silveira & Perry 1991) then the M cell density is 140 M ganglion cells/deg^2^ at 5° eccentricity. The cortical magnification factor in *M* *fascicularis* V1 at 5° eccentricity is approximately 2 mm/deg (Xing et al., 2009). Therefore, one HC in V1 cortex (0.5 mm × 0.5 mm) at 5° eccentricity corresponds to about (0.25 deg)^2^ = 1/16 deg ^2^. Then, the number of M ganglion cells that provide direct input to one HC would be 1/16 × 140, or around 9 cells. A similar calculation can be performed with the macaque LGN data of Connolly and Van Essen (1984). They provide an estimate of the magnocellular LGN density at 5° to 10° at approximately 160/deg^2^, yielding the estimate of 10 LGN cells providing direct LGN input to each HC. Counting these direct projections and the branching Magnocellular axons from neighboring HCs (Blasdel & Lund 1983; Lund et al., 1993), these results are consistent with those from Angelucci and Sainsbury (2006), who reported that, in L4, a region 300 µm in diameter is contacted by no more than 11 LGN cells, and they proposed that the numbers of LGN inputs to cortical cells are likely much smaller.

Following the optical imaging data of Obermayer and Blasdel (1993), we sought to assign to each neuron in a designated orientation domain in L4 (Figure 1A) a configuration of LGN cells that has the potential to produce two or three ON/OFF subregions aligned in the direction specified, and to do so obeying the constraints above. Another relevant fact was V1's preference for a sf at 2.5 to 3.0 c/d (DeValois et al., 1982), so that rows of LGN cells corresponding to adjacent subregions should be separated by 3/16° to 1⁄4°.

In CSY1, we started with a (regular) triangular lattice on the plane (as in Figure 1A), which we identified with visual space. Assuming that the centers of the receptive fields of ON LGN cells correspond with points in this lattice, we placed the OFF cells at the barycenters of the triangles defined by the ON lattice. Lattice spacing was chosen so that neighboring ON (resp. OFF) cells are about 0.125° apart, resulting in 10 cells per HC. We then perturbed randomly and independently each lattice point to match the retinal mosaics of M retinal ganglion cells.

**Figure 2.**
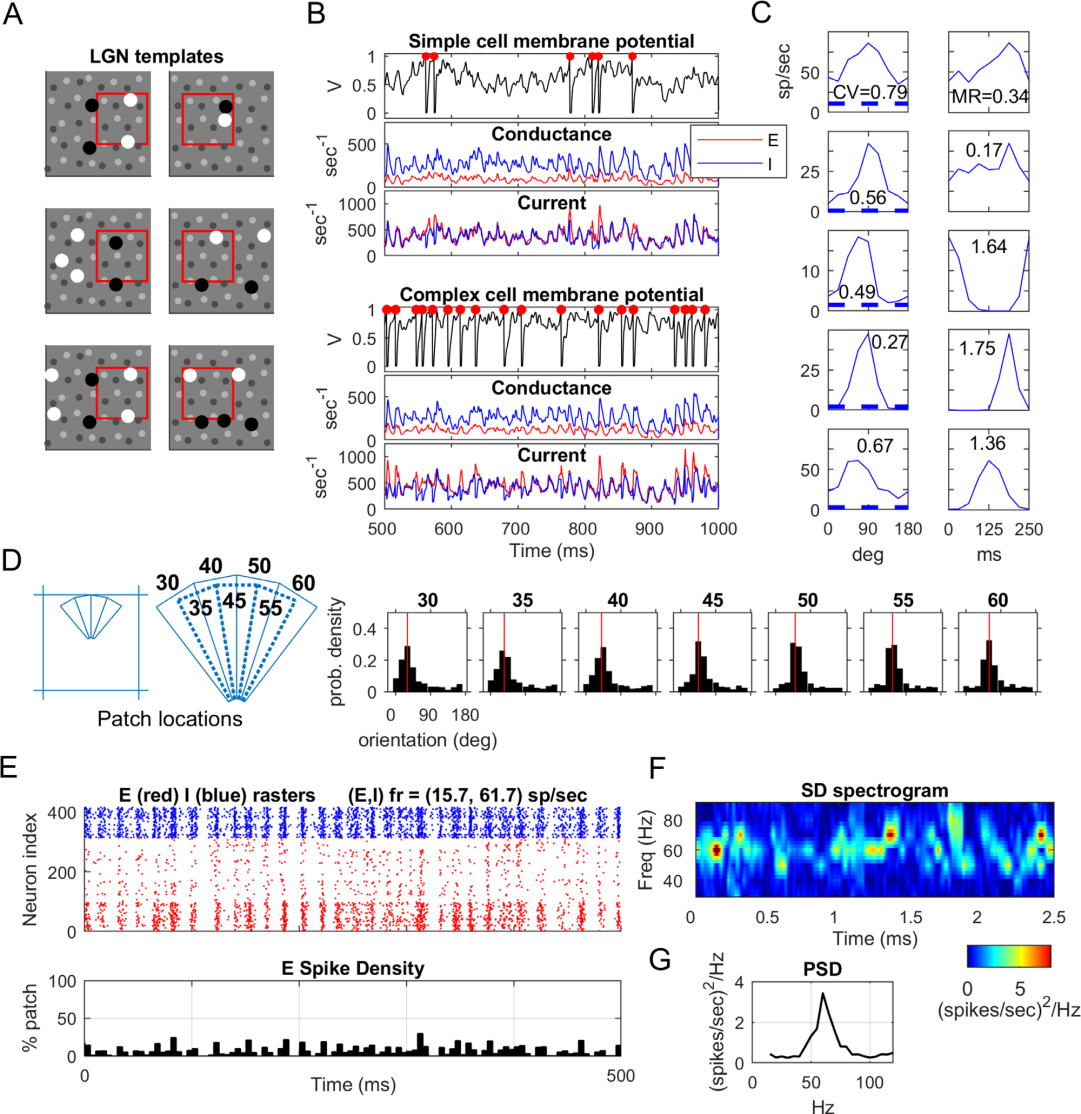
Snapshots of model outputs of CSY1. (A) A sample of LGN templates showing ON and OFF cells vertically aligned (left 3 panels) and horizontally aligned (right 3 panels). The red box corresponds to a HC. (B) Membrane potentials (normalized so spikes are fired at 1 and reset to 0) with red dots indicating spikes fired, conductance, and current traces of two model cells, simple (top) and complex (bottom), E-current in red and I-current in blue. (C) Five example model E-cells. On the left are tuning curves, the number representing the circular variance of the cell; on the right are its cycle averaged firing rates in response to a grating at 4 Hz. The numbers are modulation ratios. (D) Fine-scale orientation preferences. The central HC is divided into thin wedges (locations shown on left); the numbers are intended orientation preferences. The seven panels on the right show the angles of gratings preferred among neurons in each wedge, the red line making the intended orientation preference for the wedge. (E) Raster of neurons in a local population in response to an optimal grating at full contrast. The *x*-axis is time and the *y*-axis is neuron index; E-neurons in red, I-neurons in blue. Below the raster is a summed spike plot showing the fraction of the population spiking in 5-ms bins. (F) Spectral power as function of time and (G) power spectral density, both for same simulation as in (E).

As indicated in Figure 1A, we divided each HC of L4 into six distinct orientation domains around a pinwheel center, and stipulated that within each domain, neurons received LGN inputs favoring one of the orientations—0°, 30°, 60°, 90°, 120°, or 150°—with 0° taken to be vertical. Because the lattice we used has a three-fold rotational symmetry, it is sufficient to enumerate two sets of admissible templates, one preferring, for example, horizontal and the other 30° from horizontal. Rotating these two sets of templates by 60° and 120°, one obtains templates for the other four orientations. Permitting each cortical E-cell to have zero to six LGN inputs, arranged in two or three rows of one to three cells each, we found that there are only a small number of viable configurations. Some example LGN templates are shown in Figure 2A.

#### Layer 4Cα

We modeled nine HCs of 4Cα each measuring 0.5 × 0.5 mm^2^ . Each HC is divided into regions within which cells are intended to have similar orientation preferences. Following Beaulieu et al. (1992), we used cell densities of approximately 4,000 neurons per HC, three-quarters of which are excitatory (E) cells and the rest are inhibitory (I) cells. The I-neurons are assumed to be a homogeneous population of local circuit basket cells, a reasonable approximation for L4 (DeFelipe et al., 1999). E- and I-neurons are uniformly distributed in the model cortex, and their dynamics are governed by conductance based integrate-and-fire equations, details of which are given in the Supplementary Appendix.

The probability of a connection between model cells depends on distance and cell types (E or I), while the strength of connection is independent of distance (based on Oswald & Reyes, 2011). For presynaptic E-neurons, connection probabilities are given by Gaussians with a standard deviation of 200/√2 µm; for presynaptic I-cells, a standard deviation of 125/√2 µm (Fitzpatrick et al., 1985; Yoshioka et al., 1994). Peak connection probability between E-cells is approximately 15% on average. According to Holmgren et al. (2003) and Oswald and Reyes (2011), I-cell connections are much denser (from 50% to 100%); we set the peak connection probabilities for E→I, I→E, and I→I to be 60%. The numbers of connections and cell densities imply that, on average, an E-cell has approximately 200 E-cells and approximately 100 I-cells presynaptic to it, and an I-cell has, on average, approximately 750 E-cells and approximately 100 I-cells presynaptic to it (Figure 1B). All of these connectivity properties are the same in CSY2.

#### Feedback from L6

L4 receives substantial feedback primarily from L6 (Callaway, 1998), the effects of which are incorporated in the model. We do not model L6 as a network, modeling only its outputs as a collection of spike trains, one for each L6 neuron that projects to L4. From the cell density of L6 (Beaulieu et al., 1992) and the small fraction of E-cells that project to L4 (Wiser & Callaway, 1996), we estimated that approximately 300 L6 neurons per HC have ascending axons that terminate in L4. The axonal spreads are large (Wiser & Callaway, 1996); in the model, we assumed that most (approximately five out of six) of an L6 cell's synaptic contacts occur in a disk of radius approximately 180 µm, with some extending as far as approximately 360 µm. This gives an average of approximately 50 presynaptic L6 neurons.

#### Parameter determination

The model has more than 10 parameters, the most central ones of which are the four synaptic coupling weights S*^XY^*, where *X*, *Y* = *E*, *I*, and *X* is the target while *Y* is the source. Details on parameter determination are given in the Methods section of Chariker et al. (2016). Summarizing, we first set S*^EE^* using the fact that 10 to 20 excitatory pulses in quick succession to drive a cell across threshold places *S^EE^* between 0.02 and 0.03. From this, we determined S*^EI^* by setting the total E-current and I-current into an E-cell to be roughly balanced. We then made an assumption that *S^II^* is approximately 0.75*S^EI^, the 0.75 chosen to represent the effective lowering of *S^II^* owing to the presence of electrical coupling among I-neurons. The remaining synaptic weight parameter, *S^IE^*, was not determined by physiologic data. With the other three parameters fixed, *S^IE^* was found by numerical simulation to be the value that gave a background E-firing rate of three to four spikes per second and the corresponding I-firing rate was three to four times that.

#### Model outputs

The CSY1 model captured fairly accurately many of the basic properties of V1, including its mean and peak firing rates, as well as firing rate distributions, when optimally and orthogonally driven. Other important properties were orientation and sf selectivity, the production of simple and complex cells, and gamma-band rhythms. For the convenience of the reader, we have reproduced in Figure 2 a sample of the results to give a sense of the capabilities and performance of the model. These are only a subset of the results in (Chariker et al., 2016). In particular, we will not duplicate the results subsumed (as special cases) in the outputs of CSY2 to be reported in the Results.

Figure 2A shows templates for LGN configurations as discussed elsewhere in this article. Membrane potentials, conductance, and current traces of a simple and a complex cell are shown in Figure 2B. Here, membrane potential has been normalized so spiking threshold is at 1 and following a spike it is reset to 0. Figures 2C and D give two different views of orientation selectivity (OS). Five model E-cells are shown in Figure 2C, their tuning curves on the left and cycle averaged firing rates on the right. The numbers written in each box are circular variances (left) and modulation ratios (right). In Figure 2D, which shows fine-scale orientation preferences, we divided the central HC into fairly thin wedges that would correspond theoretically with the angles specified if one is to interpolate linearly between the 6 discrete angles represented by LGN templates. Shown in Figure 2D are the angle preferences for the neurons in each wedge. As one can see, individual neurons do not always prefer the intended orientation, although the distribution is peaked around that point.

The results of Figure 2E through 2G are taken from Chariker et al. (2018). The raster in Figure 2E depicts a gamma-band rhythm produced by a local population when optimally driven. Notice that the rhythm is irregular, and the spiking events were entirely self-organized. It was an emergent phenomenon, meaning a phenomenon that cannot be predicted by the properties of individual neurons and that occurs solely as a result of the dynamical interaction among neurons. Below the rasters are summed spike plots, showing the fraction of neurons spiking in each 5-ms bin. We show these plots to stress that gamma rhythms produced by the model are very far from full population spikes: seldom did 20% of the neurons spike together in a 5-ms period. Figure 2F shows the concentration of gamma power as function of time. As one can see, the frequencies wander irregularly through the gamma band of 30 to 90 Hz. Figure 2G shows the power spectral density of the same simulation.

### (3) The new model, CSY2

Our main motivation for constructing CSY2 was to explain contrast response. CSY1 was programmed to respond only to background and drifting gratings at full contrast; it did not have the capability to respond in a graded manner to visual stimuli of intermediate contrast as in the real V1. CSY2 is the next generation of CSY1. Its structure is shown in Figure 1A. This model inherits all of the structure above from CSY1 (except for the hand-crafted L6). Two new features were introduced. We first describe these features, followed by how the parameters were tuned.

#### A self-adjusted feedback mechanism

First we explain why CSY2 needs a dynamic feedback loop between L6 and L4. In CSY1, it was sufficient to program into the model—following data—the desired L6 spiking behavior at full contrast, because that was the only situation considered. Such a strategy cannot be applied to stimuli that vary in visual contrast, because L6 response would vary accordingly. In CSY2, we did not try to model L6 (which would be challenging given the large numbers of cell types), but rather sought to capture only the appropriate feedback L6 projects to L4. This was done by indexing L6 firing rates to the level of L4 activity around the same location around that point in time.

More precisely, we assumed, as in CSY1 (see the paragraph on L6 in the review of CSY1 elsewhere in this article), that a set of 300 E-cells in L6 per HC would project to L4. Each L6 neuron was assigned a spatial location evenly distributed throughout the HC, and each was represented by a spike train, the instantaneous rates of which were adjusted on demand according to a rule to be specified. Identifying the locations in L4 and L6, we assumed that postsynaptic to the L6 neuron at location *x* there was a set of neurons in L4, most of which lay within a radius of approximately 200 µm centered at *x* with some as far as 300 µm or more (Callaway, 1998). Each L4 E-neuron had 38 to 55 presynaptic L6 neurons; those with fewer LGN inputs (likely complex cells) had more L6 inputs. On average, each I-neuron in the model's L4 had 100 to 110 presynaptic L6 neurons.

To determine the appropriate amount of feedback the L6 neuron at location *x* at time *t* should provide, we created an *L6 response function* f(*R*) to be used as follows: We measured the mean firing rate of L4 neurons in a disk of radius 75 µm centered at *x* for a brief period of approximately 50 ms before time *t*. Call this number *R*. Then the instantaneous L6 firing rate at *x* was set to f(*R*). The choice of the function f(*R*) follows known L6 spiking characteristics: When *R* is the spontaneous firing rate of L4, f(*R*) is approximately 5 spikes/s, and when L4 is firing at peak values, f(*R*) is approximately 80 spikes/s. (The L6 firing rates are known to be quite high, with a synaptic failure rate of about 50%.) Between these two extreme values of R, we assume f(*R*) is increasing and has the shape of a sigmoidal function which is quite reasonable based on L6 firing rates (Hawken et al., 2019).

We located a function f(*R*) that produced satisfactory responses. This function does not depend on *x* or on *t*, and is not stimulus dependent. The assumption here is that L6 firing rate follows that in L4 averaged over space and time: Whatever L4 is doing, L6 sends back a slightly diffused current commensurate with the instantaneous firing rate of L4. The function f(*R*) does not determine L4 response; it specifies how much to magnify it. L4 and L6 interact continuously, negotiating their respective firing rates. We emphasize that the choice of f(*R*) does not prescribe the firing rate in L4, but that it does influence the range of responses possible in L4.

In CSY2, L6 feedback is net excitatory because in this model L6 neurons have more synaptic coupling to E-cells than to I-cells. We arranged it this way because the problem in macaque V1 is poverty of excitation, because of the very weak feedforward synaptic drive. The situation in mouse V1 seems to be quite different, with a much stronger feedforward drive and also much lower visually driven firing rates than in macaque V1 (Lien & Scanziani, 2013). There are reports about mouse V1 that indicate that L6 input to L4 is net suppressive there (Bortone et al., 2014; Olsen et al., 2012). However, the evidence about cat V1 is mixed about whether L6 feedback to L4 is suppressive or excitatory (Bolz & Gilbert, 1986; Grieve & Sillito, 1991). Suppressive L6 feedback in macaque V1 would be hard to reconcile with the sparsity of LGN input, the steep response versus contrast functions, and the higher firing rates in L4 in macaque V1.

#### Synaptic dynamics and excitability control

The features below are all known to be present in the real cortex. They were not needed for OS, the primary purpose of CSY1, but are needed in CSY2 to control the graded response of neurons to increasing contrast.

(a) *Synaptic depression of I-neurons*. I-neurons are known to become less effective when the spikes follow one another too closely (Galaretta & Hestrin, 1999; Gibson et al., 1999). To achieve this, we decreased the synaptic weight from I to E for approximately 40 ms or so after each spike: If an I-cell spikes at time *t*_1_ and it previously spiked at time *t*_0_, then
  $$S^{EI}\left(t_{1}\right)=S_{baseline}^{EI}*\left(1-0.12\right)*e^{\left(t_{0}-t_{1}\right)/20}$$
(b) *Facilitation of L6 neurons*. It has been observed empirically that at higher firing rates, L6 E-neurons produce larger excitatory postsynaptic potentials (EPSPs) in L4 neurons (Tarczy-Hornoch et al., 1999). In the model, we set its coupling weight at 60 spikes/s to be about 1.1 times that at 30 spikes/s.
(c) *Controlling excessive E-spiking in L4*. In real cortex, it is known that *K*_v_ currents in certain E-cells contribute to increased thresholds for potential generation in repetitive spiking (Yuan et al., 2005). To curb excessive excitation in L4, especially when exacerbated by positive feedback from L6, we raised the spiking threshold for E-neurons by 20% after each spike, to be relaxed back 90% of the way in 10 ms. This adaptation mechanism helped to control E-cell firing.

We view it as a strength of the model to find functional consequences of known cellular mechanisms (a) to (c).

### Parameter robustness and simulations performed

Starting from the parameters of the CSY1 model (see Chariker et al. (2016) for details), the bulk of the parameter tuning for CSY2 was done to locate a feedback response function that, together with the synaptic dynamics and control of neuronal excitability not present in the previous model, produced satisfactory firing rates in response to the full range of contrast. We did this using a small patch of the central HC of the model preferring the vertical orientation, and several drifting gratings at different contrasts, all aligned with the vertical and with a sf of 2.5 c/d. Once approximate parameters were located, we performed sweeps (varying key parameters) to select the best parameters and to establish robustness in parameter dependence.

The parameters obtained thus far were chosen to produce good results for one small patch in the model cortex and for three to four gratings as described in the last paragraph; there was no guarantee that they would perform in other situations. To confirm that the chosen parameters worked in general, we simulated the entire model's response to gratings at all different orientations, for full ranges of spatial frequencies and contrast. This three-dimensional matrix of stimuli required approximately 700 simulations each lasting 10 s (or 20–30 minutes of computation time). In each simulation, all spikes fired by each of the 36,000 neurons in the model cortex, as well as their membrane potentials and currents, were recorded and stored in a database that we queried to obtain the various kinds of information needed to produce the figures shown.

The parameters used are given in the Supplementary Appendix. The codes will be uploaded to Github after the publication of this article.

## Results

We have organized the results into three sections corresponding to the three fundamental questions asked in the Introduction: Part (1) documents the model's response to stimuli for the full range of contrast. Part (2) discusses orientation and sf tuning in relation to contrast. Part (3) presents neuronal activity maps across the cortical surface.

### Remarks on building a coherent, comprehensive model

Our model was built up gradually: It was trained to perform one group of tasks satisfactorily before new features were added. The new feature being added here is contrast response, and we wish to clarify what adding a new feature entails, because it is much more than just tweaking parameters or introducing new parameters or mechanisms to achieve this one property alone. To build a coherent model—a single model defined by a single set of parameters capable of simultaneously replicating many cortical functions (and not a different model for each function)—the following steps are necessary every time the model is upgraded: a) We must ensure that all previously established properties are retained, and b) it is necessary to investigate the relations between new and previously established features, to ensure that they covary in ways that conform with data. We strive not just to study one visual function at a time, but to build a coherent picture of visual processing. For us, then, a) means checking that all of the properties for CSY1 continued to hold for CSY2. As an example of b), OS and sf preferences were studied in CSY1 at full contrast, and adding contrast means that we must now consider how these tuning curves covary with contrast. This is what Part (2) of this Results section is about.

### (1) Response versus contrast

#### Firing rates

**Figure 3.**
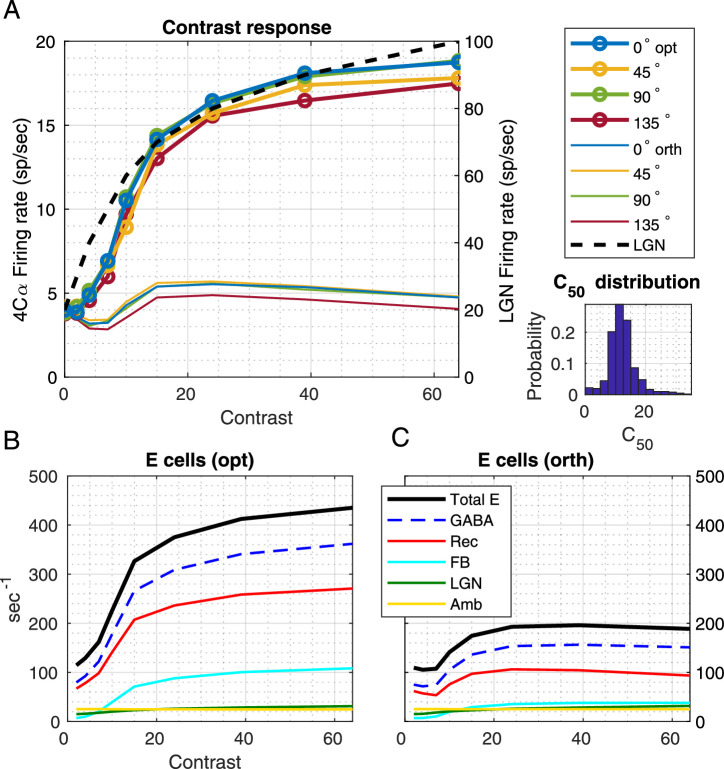
Contrast response: Firing rates and synaptic currents as functions of contrast. (A) Contrast response functions in the CSY2 model for the 0°, 45°, 90°, and 135° regions in response to a drifting grating aligned with the preference of the region and to a grating orthogonal to preferred for that region. The firing rates reported were obtained as follows: Each simulation was run for 10 s, and the mean firing rate of each neuron over the last 5 s was recorded. We then dropped the 5% to 10% of silent neurons (the peak firing rates of which were <5 sp/s for all gratings), and the firing rates shown are averages of those for the remaining neurons in the specified region. The contrast response function for the LGN input (Kaplan and Shapley, 1986; Kaplan et al., 1987) (in peak firing rates) used in the model is drawn as a dashed line. Inset shows the distribution of C_50_ for all regions combined. (B and C) Plotted versus contrast are the mean synaptic currents received by E-cells, averaged over neurons in the vertical patch over 5 s in time, in response to the vertical grating (B) and in response to the horizontal grating (C). The different curves are for recurrent excitatory current from within L4 (Rec E), Feedback from L6 to L4 (FB), LGN feedforward (LGN), ambient (Amb), and gamma-aminobutyric acid (GABA) inhibitory current (plotted in dash to indicate that this is the magnitude of a current that is opposite in sign to the other excitatory currents). Total excitatory current is plotted as a thicker black curve. The membrane capacitance was normalized to 1 (McLaughlin et al., 2000). Therefore, conductance was proportional to the inverse of the membrane time constant and has the same units, s^−1^.

The graphs in Figure 3A are population averages of firing rate versus contrast for optimal and orthogonal stimuli. A region slightly larger than the central HC in the nine HC model cortex was divided up evenly into eight patches or zones in the shape of wedges organized around the pinwheel center. For each patch, the response to a drifting grating at the optimal <orientation, sf> was computed for the full range of contrast for each neuron in the patch. The responses graphed are population averages of mean firing rate averaged over 300 to 400 excitatory neurons in each patch, and over 5 seconds of presentation of the stimulus. We excluded from the averages drawn in Figure 3A the weak responses of silent neurons, that is, neurons the peak responses of which to gratings of all orientations and spatial frequencies were below 5 sp/s, as is commonly done in the experimental literature, so that we could compare model results with data. Only 5% to 10% of neurons in CSY2 were silent neurons; this percentage in the model roughly agrees with V1 data obtained informally by Shapley and M.J. Hawken, and colleagues (unpublished results).

Results for the four patches intended to prefer 0°, 45°, 90°, and 135° are plotted in the main panel of Figure 3A. They show that the firing rate versus contrast curves for neurons with different orientation preferences have the same general shape, rise steeply and then hit a response ceiling, as in V1 data (Albrecht & Hamilton, 1982; Henry et al., 2013). Also plotted in Figure 3A are the firing rate versus contrast curves for stimuli that are orthogonal to the preferred orientation; these are the curves plotted without data points. It can be seen that the responses to orthogonal do not increase much with contrast, an indication of the OS of the model.

#### Synaptic currents

The CSY2 model, composed of conductance-based, integrate-and-fire neurons, allows us to examine the contrast dependence of excitatory and inhibitory currents in model neurons. The ability to analyze synaptic currents is crucial for understanding the mechanisms that produce realistic contrast response functions. Figure 3B shows the currents from various sources received by E-neurons in the vertical preferring patch when driven by a vertical grating; the currents, averaged over the nonsilent neurons in the patch (as in Figure 3A) and averaged over time (5 s), are shown as functions of contrast. What can be gleaned from these plots is that all currents increase with contrast, and that recurrent L4 excitation is the largest component of the E-current, the second largest source being that from feedback. In Figure 3B and 3C, the absolute value of the inhibitory synaptic current is plotted, to facilitate the comparison of E- and I-currents. We have used a dashed line for the inhibitory current to remind the reader that this current has a negative sign. (Please note that we used s^−1^ as the units of current, and conductance, as in previous articles such as Chariker et al. (2016), (2018). The reasoning behind this convention for current and conductance units is given in the Supplementary Appendix section 0.2, second paragraph.)

At peak contrast, L4, L6, and LGN provide 60% or more, 25%, and a little less than 10%, respectively, of the total E-current in E-cells (Fig. 3B), with the rest coming from ambient (representing other modulating effects not modeled). The same plot also shows that neuronal interaction within L4 produces more inhibitory synaptic current (blue dashed) than excitatory (red). However, when other sources of excitation (L6, LGN, and ambient) are included, then the total E-current (thick black) exceeds I-current at all contrasts.

Figure 3C shows synaptic currents in the same vertical-preferring group of E-cells in response to a horizontal (orthogonal) grating. The mean LGN currents for the situations in Figures 3B and 3C are identical, because neurons in optimal and orthogonal patches receive the same number of impulses per second from LGN cells. Only their input timing differed (there is much more spike rate modulation at the preferred orientation as noted by others [Troyer et al., 1998]), and it is this timing difference that is responsible for initiating the OS evident in Figure 3A and in later figures.

In CSY2 as in CSY1, as well as in the real cortex, there are no more than a handful of afferent LGN cells driving each 4Cα neuron. As a consequence of the very sparse LGN input to V1, the change in total LGN current as stimulus contrast increases from background to full contrast is quite small (Figures 3B and 3C). It is remarkable that this small increase in Excitatory current sets off the full range of firing rate versus contrast (Figure 3A). As stated in the Introduction, one of the fundamental questions about contrast response is: How exactly does this work; how do cortical neurons follow the (rather subtle) changes in such small feedforward LGN input?

#### Analysis: Cortical mechanisms contributing to the large amplification of weak LGN inputs

**Figure 4.**
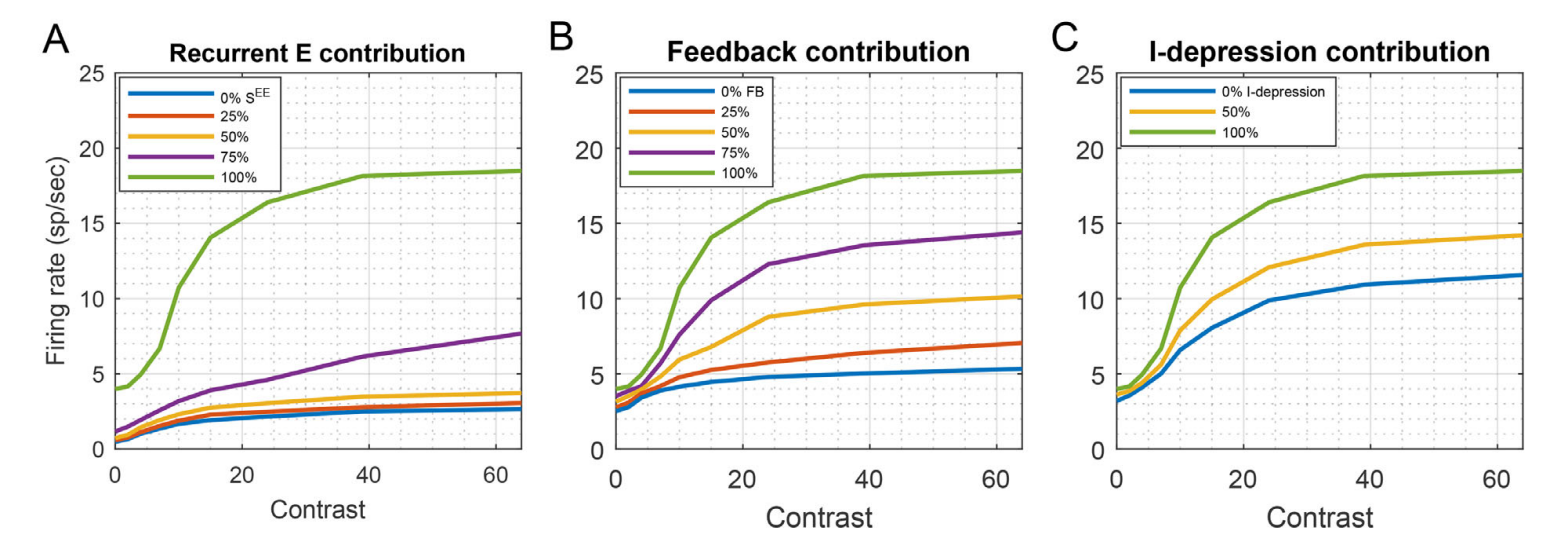
Factors that influence model contrast response. Panels A–C show three factors that contributed to the steep increase of the contrast response function. (A) The contribution of recurrent excitation within L4. The plots show mean firing rates in the vertical patch when driven by vertical gratings at various contrasts, with the synaptic coupling S^EE^ between E-cells in L4 systematically decreased to 0%, 25%, 50%, and 75% of the value used in the CSY2 model; all other parameters were unchanged. Contrast response collapsed even with 75% of the recurrent excitation in the CSY2 model. (B) The contribution of feedback from L6. As before, all other parameters were unchanged except for the amount of feedback. (C) The contribution of I-cell synaptic depression.

Having demonstrated that the CSY2 model produced desirable responses to contrast (Figure 3), we now use the model to investigate how cortical cells follow the small changes in LGN currents. We found that L4 firing rate bootstraps itself up through the combined effects of many different neural mechanisms. To understand the effect and relative importance of each, we have isolated them in Fig 4.

(i) First and foremost is recurrent excitation within L4. As demonstrated in Figure 4A, the effects of decreasing S^EE^, the synaptic coupling strength between E-neurons, were catastrophic, underscoring the role of recurrent excitation in the model's contrast response. For instance, when the synaptic weight of recurrent excitation, S^EE^, was decreased by 25%, the firing rate at high contrast was decreased from 18 sp/s down to 8 sp/s; when S^EE^ was halved, the firing rate at high contrast was only 4 sp/s. Note that in Figure 4A, when recurrent excitation was reduced to 0, the feedforward LGN input in CSY2 was so weak that it produced a firing rate of only 3 sp/s at the highest contrast.
(ii) Next in importance was the positive feedback between L4 and L6 (Figure 4B). When the strength of feedback was halved, cortical spiking was less than 10 sp/s.
(iii) Next in significance was synaptic depression of I-cells (Figure 4C), which is instrumental in the steep rise of the contrast response curve. The presence of such depression has been clearly documented in experimental data (Galaretta & Hestrin, 1999; Gibson et al., 1999).
(iv) Finally, facilitation of the excitation of L4 neurons by L6 cells (Tarczy-Hornoch et al., 1999) also played a role, bringing the contrast response of L4 to saturation at midcontrast of about 30% (not shown).

CSY2 has thus identified contrast response as a functional consequence of the combined effects of known cellular mechanisms like synaptic depression in I-cells, and facilitation of L6 feedback.

### (2) Orientation and sf tuning as functions of contrast

**Figure 5.**
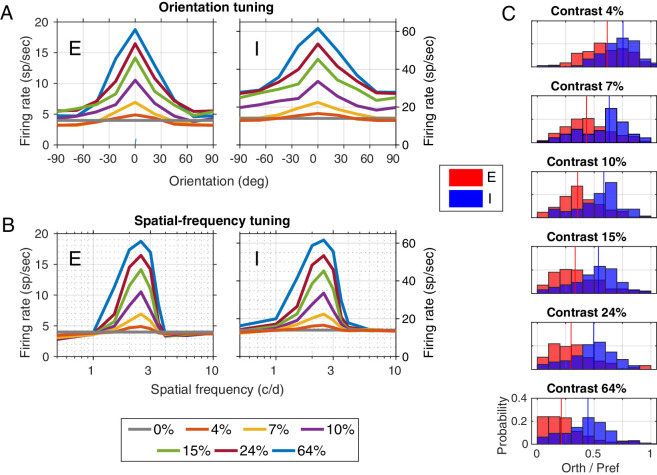
Tuning for orientation and sf for various contrasts. (A) Orientation tuning curves. Gratings in eight orientations evenly spaced from –90 to +90° with sf 2.5 c/d were presented at seven different contrasts, and mean firing rates of the vertical patch (0°) are shown; E-cells (left), I-cells (right). (B) The sf tuning curves. Gratings aligned with the orientation preference of the population at 10 different sf and seven different contrasts were used. Again, E-cells (left); I-cells (right). (C) Histograms showing orthogonal-to-preferred firing rates at various contrasts, for neurons in the vertical patch. Distributions for E-cells (red) and for I-cells (blue) are overlaid.

**Figure 6.**
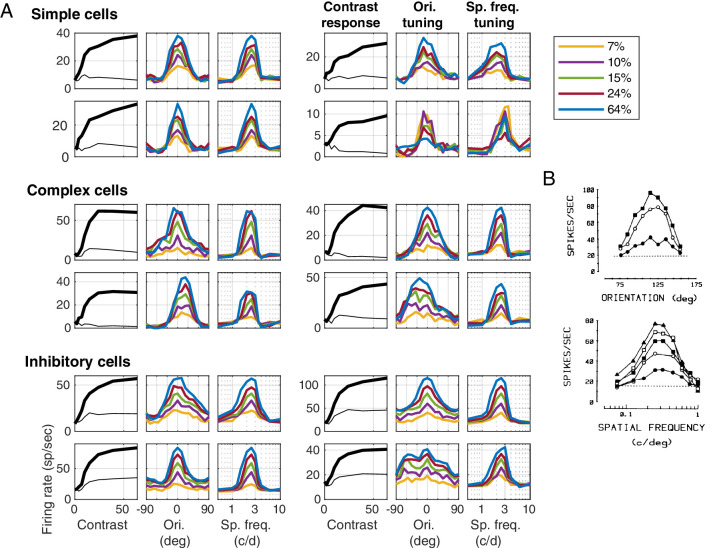
Individual cell data. (A) Contrast response curves, orientation and sf tuning of 12 model cells, four simple excitatory, four complex excitatory, and four inhibitory, all taken from the vertical patch (used in Fig 5A, B, C). (B) Orientation and sf tuning data at different contrasts from cells in cat V1 from Skottun et al. (1987), reproduced with permission.

As explained at the beginning of the Results section, to develop a coherent picture of the cortex it is necessary to go beyond studying cortical functions one at a time: we need to understand how they covary. With this in mind, we challenged the model with a 3-parameter family of gratings varying in orientation, sf, and contrast*.* CSY2 is able to simulate population data about spatial tuning for visual grating patterns. The model does this over the full range of contrast without any adjustment of parameters at different contrasts (Figures 5,6).

Population-averaged tuning profiles of firing rate versus orientation are provided in Figure 5A, and the corresponding tuning profiles of firing rate versus sf are provided in Figure 5B. First, consider orientation tuning of model E-cells (Figure 5A, left). The response at the preferred orientation increases while the response at orthogonal changes little with increasing contrast. Therefore, the OS of E-cells (indexed by the ratio of the response of orthogonal to preferred orientations) increases at higher contrast (Figure 5C). Remarkably, the model is in quantitative agreement with V1 data on this crucial point (Alitto & Usrey, 2004; Johnson et al., 2008; Skottun et al., 1987) as illustrated in Figure 6B, where data for cat V1 from the original Skottun et al. (1987) article are reproduced. Model I-cells, in contrast, show much less increase in OS with increasing contrast (Figure 5A, right, and Figure 5C).

Tuning profiles for firing rate versus sf (Figure 5B) show a similar trend: sf selectivity (the preference for certain optimal frequencies) increases at higher contrast. The sf tuning dependence of E-cells on contrast is consistent with real V1 (Figure 6B).

Neither the model nor the real cortex shows precise contrast invariance of orientation tuning or sf tuning. Rather, what is seen is only an approximate invariance in the responses of E-cells: The growth of preferred responses with increasing contrast and the clamping of responses to nonpreferred stimuli to low levels at all contrasts. A functional consequence of this is increase of feature selectivity in E-cells with increasing contrast.

The model agrees with experimental data on how much OS is increased at higher contrast. This test of the model's predictive power is important. We show in Figure 5C the distributions of orthogonal-to-preferred firing rates (O/P) for E-cell and I-cell populations in the vertical patch as functions of contrast. Of note is the magnitude of the leftward shift of the O/P distribution for E-cells between 10% and 64% contrast, the range explored in experiments. It is approximately 0.2, a value that is comparable to the decrease in O/P ratio, or circular variance, (increase in OS) found in experiments (Alitto & Usrey, 2004; Johnson et al., 2008). Thus, the CSY2 model accounts quantitatively for the contrast dependence of OS found in experiments on populations of V1 neurons.

#### Single cell results

Results from examples of model neurons are shown in Figure 6 to illustrate both the diversity and the trends underlying the population averages—and also for comparison with electrophysiologic data. Firing rate versus contrast, and orientation and sf tuning curves are plotted for excitatory and inhibitory neurons taken from the same vertical patch used in Figure 5.

#### Analysis: Mechanisms for increased selectivity at high contrast

First we explain why in the model responses of E-cells to optimal stimuli grow more with increasing contrast than responses to nonpreferred stimuli, answering the second question posed in the Introduction. Being more broadly tuned (Nowak et al., 2008), I-cells when stimulated supply a minimum level of suppression across the entire model cortex. This minimum level of suppression extends over all orientations and beyond the ranges of sf to which E-firing responds (Figure 5), forming a shield of suppression that serves as a barrier to increasing E-activity in many simulations; additional thrust is required to break through the shield, but once it has broken through, E-activity increases more easily owing to recurrent excitation and positive feedback with L6. For preferred stimuli, this breakthrough is enabled by the boosting of E-cell activity through the combined action of the neural mechanisms discussed in connection with Figure 4. For nonpreferred stimuli, the E-cell firing rate is kept down by the broad shield of I-cell suppression. This is the mechanism by which the model increases its feature selectivity at high contrast.

But what brought about the broader tuning of model I-cells in the model? One reason is that S^EI^, the inhibitory synaptic coupling weight from I-cells to E-cells, is higher than S^II^, the inhibitory synaptic coupling weight from I-cells to I-cells. We used a smaller value of S^II^ to compensate for the effects of electrical coupling (which was not modeled) among pairs of I-cells (Galaretta & Hestrin, 1999; Gibson et al., 1999). Lower S^II^ leads to a higher operating point for I-cells than E-cells, causing them to fire spikes even for nonpreferred stimuli (Figure 5A and 5B). A secondary reason is that LGN affects E- and I-cells differently, producing larger EPSCs in I-cells—a property of the model that follows data (Beierlein et al., 2003).

### (3) Activity maps across the cortical surface

Because the bulk of the excitatory current for cortical neurons comes not from LGN but from intracortical interactions (Figure 3B and 3C), studying spatial maps of cortical activity on the cortical surface helps us to understand cortical function. In CSY2 as in real cortex, when the contrast of a grating is increased, firing rates of cells in optimally driven domains climb steeply, while firing rates of cells in orthogonal domains are virtually unchanged (Figures 3 and 5). Within an HC, these domains are no more than approximately 100 µm apart. This means that, at high contrast, the firing rate profile across the cortical surface is extremely uneven, with tall peaks separated by deep valleys. It is intriguing how such a wildly varying landscape of activity in mutually interacting neurons can be stable across contrast. To answer this question, we present in this last part of Results simulated activity maps in CSY2 analogous to fMRI images (as in e.g., [Kay et al., 2013]) and optical imaging (Carandini & Sengpiel, 2004; Obermayer & Blasdel 1993), except that the activity depicted is firing rates and the resolution can be as close to the neuronal level as one wishes.

#### Cortical activity in response to gratings of different orientations

**Figure 7.**
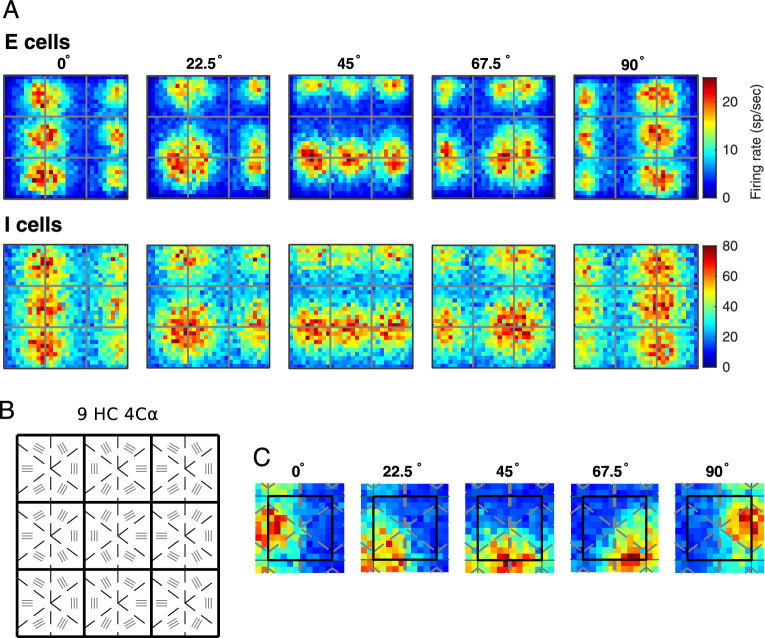
Orientation preference maps in the model. (A). Activity maps of E-cells (top) and I-cells (bottom) in L4 in response to five gratings at 64% contrast; orientations of the gratings are written above the panels. Each panel is composed of 900 pixels, each one of which corresponds to a 50 × 50 µm^2^ patch of cortex containing about 30 E-neurons and 10 I-neurons. The color represents the mean firing rate (see color bars) averaged over the neurons in a pixel and over 1 s in time. (B) To facilitate the interpretation of the activity maps in (A), we have replotted from Fig 1 the L4 component of the model showing the locations of intended orientation domains in the nine HCs. The six regions in each HC, arranged counterclockwise in a pinwheel configuration, prefer 0°, 30°, 60°, 90°, 120°, and 150°, vertical labeled as 0°. Neurons in these regions are given six distinct sets of LGN templates. (C) Highlighting of activity of E-cells in the central HC (region enclosed in black square) in response to the five orientations used in (A).

The activity maps in Figure 7 were produced in simulations of V1's response to drifting sine gratings of high contrast.

Consider first the activity maps of E-cells (Figure 7A, top row). Looking at the intended orientations map in Figure 7B, we see that the vertical preferring region is composed of three diamonds stacked up vertically on the left side and three half-diamonds on the right side of the nine HC model cortex. This means that when the vertical, or 0°, grating is presented, one should expect these diamond-shaped regions to respond most vigorously. The top left panel of Figure 7A shows that this is indeed the case. The other four panels show cortical responses to four other gratings. The reader can check, by comparing with Figure 7B, that in each case the regions that responded most vigorously are those whose designated orientation preferences aligned with the grating.

Observe also that the regions of elevated spiking in each of the panels in fact spill over the edges of the designated orientation domains. This finding is consistent with the fact that neurons do not respond only to a single orientation; the amount of spilling reflected the breadth of the orientation tuning of neurons.

Activity maps for I-cells look different. Figure 7A, second row, shows activity maps for I-cells. Observe that i) E- and I-cells are co-activated in the sense that their firing rates are elevated in roughly the same regions of the model cortex for each grating presented, and ii) I-firing rates in these regions are three to four times higher than those of E-cells, consistent with data. Also, iii) the regions of elevated I-spiking are larger than those for E-cells, and I-firing rates are greater than 20 to 30 spikes/s virtually everywhere in the model cortex, unlike E-cells, which in orthogonal regions fire at or slightly above background level, that is, at approximately 4 spikes/s (cf. Figure 5A).

Figure 7C zooms in on the response of E-cells in the central HC for the same five gratings. Here one can see clearly that over the 1-s period used for the time average, firing rates vary considerably from one pixel (corresponding to about 30 E-neurons) to the next, but that overall, the regions of elevated spiking rotated smoothly around the pinwheel with the grating's orientation.

#### Activity maps versus contrast

**Figure 8.**
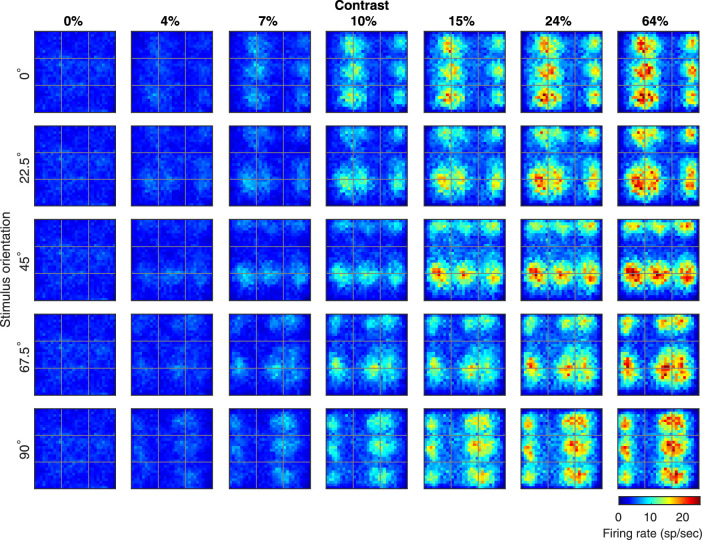
Activity maps at different contrasts in the V1 model. These are activity maps of the same type as Figure 7 showing firing rates in the nine HC model cortex in response to 2.5 c/d gratings at various orientations and contrasts. Each row represents the responses to a drifting grating of a particular orientation; each column a different contrast. Contrast increases from left to right. Firing rates are given by the color bar, and resolution of the maps is as in Figure 7.

Orientation preference maps are usually measured in optical imaging experiments with high contrast stimuli, as in Figure 7. To investigate whether such maps are stable with contrast, we ran simulations, the results of which are presented in Figure 8. The rightmost column in Figure 8 is identical to the top row in Figure 7, and each row in Figure 8 from left to right shows increasing contrast for a grating of the same orientation. Figure 8 shows that, in CSY2, activity maps are very stable with contrast: The regions of increased activity in each row have nearly identical footprints on the cortical surface starting from very low contrast. That is, as contrast is increased, steady-state firing rate profiles, varied in shape and in magnitude as they are across the cortical surface, are robustly maintained. These results are consistent with the optical imaging results of Carandini and Sengpiel (2004).

Below we explain the dynamical considerations behind these activity maps, and how they are impacted by the various mechanisms involved.

#### Analysis: Balancing excitation and inhibition across the cortical surface

Each of the panels in Figures 7A and 8 represents a continuum of local equilibria, obtained through the dynamic balancing of excitation and inhibition, across the surface of L4 in response to a visual stimulus. In the CSY2 model as in real cortex, the circuitry of L4 is roughly spatially homogeneous. The inputs received, in contrast, vary from point to point depending on the stimulus, and the patterns of activity seen are produced by these (rather subtle changes in) inputs together with corticocortical interaction.

We elaborate on this point, because it is an important difference between our model and models in which a transducer function converts each neuron's feedforward input into spikes. In CSY2 as in real cortex, the bulk of the excitatory current received by a neuron is from within cortex, with only a small percentage of the current coming from the LGN (Figure 3B). At each location—think of it as a pixel in the activity maps in Figures 7A and 8—instantaneous rates of spike firing are determined by balancing the inhibition received by the local population against the excitation from four sources: i) LGN, ii) E-neurons from L4, iii) feedback from L6, and iv) ambient sources representing other modulatory forces. According to the leaky integrate-and-fire equation (Supplementary Appendix), excess excitation over inhibition controls spike firing. The E- and I-input to each neuron is summed over fairly large areas, over many neighboring pixels, determined by the axonal lengths of neurons (Figure 1B). The E/I balance point differs from pixel to pixel because visual stimuli typically cause the values of the E- and I-inputs to be location dependent. Each of the activity profiles shown depicts a nonequilibrium steady state, representing negotiated local equilibria that hold simultaneously at all pixels everywhere in the model cortex.

The presence of peaks and valleys in close proximity in the activity maps are an illustration of the very delicate balancing of E- and I-input from the network to each neuron in the network. Consider the response of L4 neurons in vertical and horizontal-preferring domains of the same HC. As contrast is increased, both excitation and inhibition are increased (e.g., Figures 3B and 3C). Excess excitation over inhibition, however, is larger in the optimally driven domain due to recurrent excitation enhanced by feedback and synaptic depression of I-neurons (see text accompanying Figure 4). There is also the broader inhibition that sets a threshold that is exceeded in optimally driven domains at high contrast, but not in orthogonally driven domains (see the Analysis in Results (2)). At each contrast, the nonequilibrium steady-state solution smoothly interpolates the peaks and valleys in spiking activity in the different orientation domains.

## Discussion

The CSY2 model accurately simulated V1 experimental data, producing the following three outstanding results: 1) It simulated the firing rate versus contrast function observed in V1 neurons (e.g., Albrecht & Hamilton, 1982, and many others afterwards); 2) it simulated the contrast dependence of orientation tuning, with stronger selectivity at higher contrast, agreeing with monkey, cat, and ferret V1 data (Alitto & Usrey 2004; Johnson et al., 2008; Skottun et al., 1987); 3) it produced maps of cortical activity across the surface of V1 that were stable with contrast as was reported in V1 optical imaging experiments (Carandini & Sengpiel, 2004). We hypothesize that the mechanisms in CSY2 that produced these three results are likely to be responsible for the corresponding phenomena in real cortex.

### Comparison with previous models

The way the CSY2 model achieved its results was different from that of all previous visual cortex models. Many previous models were built with explicit or implicit assumptions that excitatory synaptic input from the LGN was sufficient to cause the cortical cells to spike (Goris et al., 2015; Persi et al., 2011; Tao et al., 2004; Troyer et al., 1998). This is very different from the CSY2 model, in which LGN excitatory input is quite weak. In CSY2, corticocortical excitation provoked by LGN input is what breaks through the shield of inhibition to cause spike firing. No previous model tested whether such large corticocortical amplification of weak LGN input could be made to work in a large-scale simulation of cortical function. The success of CSY2 in simulating cortical function means that the cellular and network mechanisms incorporated into CSY2 are enough to explain the major phenomena of the cortex's contrast response.

As to how CSY2 compares with the normalization model (Carandini & Heeger, 2011) developed to explain data on the cortical contrast gain control (Ohzawa et al., 1982), the idea of normalization was intended to provide insight into the way cortical inhibition could regulate many disparate cognitive functions. It is a descriptive model not constrained by data on cortical anatomy or physiology and it was not intended to inform about the roles of cortical mechanisms in explaining contrast phenomena. Another theoretical idea is the stabilized supralinear network (SSN) theory in (Rubin et al., 2015). CSY2 takes a different approach: It is a neuroanatomy-based, biologically constrained model built for purposes of shedding light on cortical mechanisms.

There were previous models that included the idea of recurrent excitation as a mechanism for feature selectivity in V1. In particular, the large-scale model in Somers et al., (1995) was very influential in persuading theorists that recurrent excitation could play a role in the V1 OS. Somers et al., (1995) also predicted the shape of the V1 contrast response function. But the Somers et al., (1995) model was much more feedforward than CSY2 and its cortical cell density was much lower than in the real V1. The importance of recurrent circuitry in explaining contrast response is indicated more convincingly in CSY2, a model with closer resemblance to the real V1 in neuroanatomy and which explains coherently many other aspects of cortical function.

### Relation to experimental results: Predictions and postdictions

The CSY2 model has enabled us to make many new testable predictions; it also confirms and explains a number of experimental observations. Here we collect and summarize the main new model predictions and postdictions.

• *Intracellular E-* *and I-currents:* Our model results predict that a) inhibitory synaptic current tracks total excitatory current across contrast, but that b) E- and I-currents are not exactly balanced, with E-current leading (c.f., the homogeneously connected models of van Vreeswijk & Sompolinsky 1996), and c) the excess of E-current over I-current, which promotes spike firing, is correlated with the neuron's firing rate. See Figure 3B and 3C for supporting simulations.
• *Mechanisms for contrast* *response:* It has been suggested in Somers et al., (1995) that recurrent excitation played an important role. Here we amplify this assertion: Our analysis (Figure 4A) showed that a 25% decrease in S^EE^ decreases contrast response to no more than a few spikes above the spontaneous level. Another model prediction is that the dynamic positive feedback loop between L6 and L4 is an essential mechanism for contrast response (Figure 4B).
• *Net excitation of feedback from L6:* L6 has been shown to be net-inhibitory in mouse V1. For monkey, whether the L6 projection to L4 is net excitatory or net inhibitory has yet to be determined. Our model predicts that, owing to the paucity of LGN input in macaque V1, for L4 neurons to produce the observed firing rates (which are much higher than those for mouse), it is necessary that L6 input to L4 be net excitatory in macaque V1.
• *Broader tuning of I-cells:* One of the postdictions of our model is that I-cells are more broadly tuned for orientation than E-cells, especially at high contrast; we offered mechanisms for this phenomenon, and connected it to stronger feature selectivity at high contrast (Results (2)). We predict also that future imaging results will show, when E and I-responses are separated, that I-cells will have a larger footprint of elevated spiking on the cortical surface than E-cells (Figures 5 and 7).
• *O/P ratios as a measure of* *OS:* The amount that contrast enhances OS can be quantified by the O/P ratio which for E-cells is predicted to decrease by 40% between just suprathreshold contrast and high contrast (Johnson et al., 2008). Our model results are in close quantitative agreement with this observation. As for I-cells, no data are available, and our model predictions are that their O/P ratio will decrease with increasing contrast, although not by as much as for E-cells (see Figure 5C).
• *Diversity of neuronal responses:* Our model produces—through dynamical interaction of neurons—greater amounts of diversity in E-cell firing rates in contrast response and in OS and sf selectivity (Figures 3A, 5, and 6) than most previous models, in agreement with data.
• *Stability of neuronal activity maps with contrast:* The CSY2 model reproduces activity maps similar to the images in, for example, Kay et al. (2013) and Carandini and Sengpiel (2004), but with much higher spatial and temporal resolutions, down to real-time interaction on the neuronal level. CSY2 predicts that such activity maps are stable across the full range of contrast.

### Greater emphasis on population activity in model analysis

Another feature that sets this article apart is that it has a stronger emphasis on the analysis of CSY2's population activity (Figures 3, 5, 7, and 8) than previous model analyses. Although individual neuron properties (Figure 6) in models are useful for comparison with data from electrophysiology, cortical mechanisms such as recurrent excitation and indirect suppression can and do emerge from the dynamic interaction of neurons, as a result of which population responses can be quite different from those from individual neurons. Because cortical function is likely influenced as much if not more by population responses as by attributes of individual neurons, we believe there is a need to devote more effort to understanding population dynamics in computational modeling.

Examples of the analysis of population responses are the activity maps in Figures 7 and 8. CSY2's production of these maps required the balancing of excitation and inhibition in L4 from multiple sources: Feedforward input from the LGN, feedback from L6, and the synaptic inputs the neurons in L4 receive from lateral interaction from within this layer. This had to be done not just for a single local population, but simultaneously for all local populations in the model cortex, each local population receiving different inputs owing to the different ways it was impacted by the visual stimuli. It is important to remember that all the images in Figures 7 and 8, as for all of the figures in the article, were produced using a single set of equations and a single set of parameters; it was only the input light intensity maps that differed (see Methods (1)).

Achieving dynamic balance with desired outcomes for a significantly larger set of circumstances than the number of adjustable parameters is both a goal and a high bar for computational modeling. This is what we have demonstrated for CSY2 with the activity maps in Figures 7 and 8. These maps offer a new and useful view of cortical population dynamics quite different from those obtainable through current experimental techniques.

## Supplementary Material

Supplement 1
