# Supplementary material for: Contrast response in a comprehensive network model of macaque V1
Source: J Vis. 2020 Apr 24;20(4):16. doi: 10.1167/jov.20.4.16 (PMC7405768; doi:10.1167/jov.20.4.16)
Supplement: Supplement 1 [file jovi-20-4-16_s001.pdf]

Appendix

We model a 1.5×1.5 mm^2 patch of macaque V1 layer 4Cα with a network of integrate-and-fire point neurons. The neurons are divided into 9 hypercolumns (HC's) with 3000 excitatory (E) neurons and 1000 inhibitory (I) neurons per HC. Additionally, 250 magnocellular LGN cells and 3000 layer 6 neurons are modeled to provide input to $4C\alpha$. LGN is driven by drifting grating stimuli which can vary over contrast, orientation, and spatial frequency, all with a temporal frequency of 4 Hz.

Here we provide a summary of the equations governing neuronal dynamics in the model. The equations for LGN are given in section 0.1, and the basic equations for 4Cα in section 0.2. Modifications to the 4Cα equations are provided in section 0.3, and the equations involved in simulating layer 6 feedback are provided in section 0.4. Model parameters not directly determined from data are listed in section 0.5.

0.1 Dynamics of LGN cells

Following Lin et al. (2012), we modeled the dynamics of individual LGN cells by the integrate-and-fire equation

$$V'(t) = -cV(t) + I^{\pm}(t) + S_{\text{noise}} \sum_{i=1}^{\infty} S_i \delta(t - t_i)$$

where V is membrane potential, $c = 100$ is the leak rate (t is in sec), I^+ and I^- are deterministic currents entering ON and OFF LGN cells respectively, S_{noise} is the coupling coefficient of a Poisson-timed noise term, and t_i are the arrival times of the Poisson noise inputs. When V reaches a threshold $V_{\text{thresh}} \sim 1$, the potential is reset to 0, and a spike is sent to all postsynaptic cells in 4Cα. For a sinusoidal grating drifting in the direction u (unit vector) with temporal frequency w, the current $I(t,x)$ into an LGN cell the center of whose receptive field (RF) corresponds to location x in visual space is given by

$$I^{\pm}(t,x) = I_0(1 + \varepsilon C(|u|)\sin(p(t,x)))$$

where $I_0 = 100$ (as in Lin et al., 2012), ε is contrast, $C(u)$ is the spatial frequency response function encoding the LGN firing rate's dependence on spatial frequency of the grating ($C(u)$ was modeled as a standard Difference-of-Gaussians [Enroth-Cugell and Robson, 1966] inherited from the M retinal ganglion cell that drove each Magnocellular LGN neuron, as in Zhu et al., 2009). The phase at location x is given by $p(t,x) = wt + \langle x, wu \rangle$ (where $\langle , \rangle$ is the inner product). Constants are chosen so that background firing rate of an LGN cell is ~ 20 spikes/sec, and when driven by a grating at full contrast, peak firing rate is ~ 100 spikes/sec (Hawken et al., 1996). The contrast-response function of the LGN was simply modeled as the amplitude vs contrast from the measured values in macaque M ganglion cells (Kaplan and Shapley, 1986) and LGN Magnocellular neurons (Kaplan et al., 1987).

0.2 The basic integrate-and-fire equations for 4Cα neurons

$4C\alpha$ neurons are modeled as conductance-based, integrate-and-fire point neurons; the membrane potential V of each evolves according to

$$C\frac{dV}{dt} = -g_L(V - V^L) - (g_{\text{AMPA}} + g_{\text{NMDA}} + g_{\text{amb}})(V - V^E) - g_{\text{GABA}}(V - V^I) \tag{1}$$

where C is membrane capacitance; g_L is the leak conductance; g_{AMPA} and g_{NMDA} are the time-dependent, excitatory conductances corresponding to AMPA and NMDA receptors; g_{amb} is a conductance term representing the modulating effects of multiple substances not modeled; g_{GABA} is the time-dependent inhibitory conductance

from GABA_A receptors; and V^L, V^E, and V^I are the leak, excitatory, and inhibitory reversal potentials. The equations governing the time evolution of the conductances are given below. For simplicity the voltage is rescaled so that $V^L = 0$ and the spiking threshold is at $V = 1$. Capacitance is also moved to the RHS and absorbed in the conductances. As a result, instead of using equation (1) directly, we rewrite it as

$$\frac{dV}{dt} = -\frac{1}{\tau_\text{L}}V - (g_\text{AMPA} + g_\text{NMDA} + g_\text{amb})(V - V^E) - g_\text{GABA}(V - V^I) \tag{2}$$

where, in rescaled units, $\tau_\text{L} = 20$ ms for E cells and 15 ms for I cells (Beierlein et al., 2003), the spiking threshold is $V = 1$, upon spiking V is immediately reset to 0, $V^E = 14/3$ and $V^I = -2/3$ (Koch, 1999). Following a reset after each spike, V is kept at 0 for a refractory period of 2 ms for E cells and 1 ms for I cells. When the refractory period is over, the evolution of V resumes according to equation (2).

A remark on units: Membrane capacitance was normalized to 1 (McLaughlin et al., 2000). Therefore, conductance was proportional to the inverse of the membrane time constant and has the same units, sec^{-1}. A normalized conductance of 1000 sec^{-1} was equivalent to ~ 20 nS. Membrane current was conductance scaled by the difference between membrane potential and the reversal potential for the particular conductance channel. The currents also were calculated in normalized units where, for current, sec^{-1} is equivalent to ~ 300 pA.

To state the conductance equations, we introduce the following notation. The synaptic conductance terms g_AMPA, g_NMDA, and g_GABA are driven by the spiking activity of presynaptic cells. For neurons of type $Q \in \{E, I\}$ and $Q' \in \{E, L6, LGN, amb, I\}$, we denote by $S^{QQ'}$ the synaptic strength of a spike when the presynaptic neuron is of type Q' and postsynaptic neuron is of type Q. The ambient conductance g_amb is driven by a Poisson-timed train with rates ρ^E_{amb} for E cells and ρ^I_{amb} for I cells. The time courses for EPSC's and IPSC's are denoted by functions $G_\text{AMPA}, G_\text{NMDA}, G_\text{GABA}$ and G_amb, which are given below, following the conductance equations. The model parameters ρ^E_amb, ρ^I_amb, and $S^{QQ'}$ are given in section 0.5. For a layer 4 neuron of type Q, the equations governing the time-evolution of its conductances in equation (2) can now be stated as follows:

$$\begin{aligned}
g_\text{AMPA}(t) &= S^{QE}\sum_{i=1}^{\infty}\sigma^Q_\text{AMPA}G_\text{AMPA}(t - t_i^\text{E}) + S^{QL6}\sum_{i=1}^{\infty}\sigma^Q_\text{AMPA}G_\text{AMPA}(t - t_i^\text{L6}) \\
&\quad + S^{QLGN}\sum_{i=1}^{\infty}G_\text{AMPA}(t - t_i^\text{LGN}); \\
g_\text{NMDA}(t) &= S^{QE}\sum_{i=1}^{\infty}\sigma^Q_\text{NMDA}G_\text{NMDA}(t - t_i^\text{E}) + S^{QL6}\sum_{i=1}^{\infty}\sigma^Q_\text{NMDA}G_\text{NMDA}(t - t_i^\text{L6}); \\
g_\text{amb}(t) &= S^{Qamb}\sum_{i=1}^{\infty}G_\text{amb}(t - t_i^\text{amb}); \\
g_\text{GABA}(t) &= S^{QI}\sum_{i=1}^{\infty}G_\text{GABA}(t - t_i^\text{I}).
\end{aligned}$$

Here t_i^E are the arrival times of E spikes from all neurons in layer 4Cα presynaptic to the neuron in question; t_i^L6 are the arrival times of E spikes from all neurons in layer 6 presynaptic to the neuron in question; t_i^LGN, t_i^amb, and t_i^I are defined analogously. For both L4 and L6 E spikes, σ^Q_AMPA and σ^Q_NMDA are the proportions of presynaptic spike strength weighted to AMPA and NMDA, respectively, so that $\sigma^Q_\text{AMPA} + \sigma^Q_\text{NMDA} = 1$. We use $\sigma^E_\text{AMPA} = \sigma^I_\text{AMPA} = 0.2$ (Rotaru et al., 2011).

As for the conductance time-courses, for $R =$ AMPA, NMDA, GABA or amb, the time courses are

$$G_R(t) = \frac{1}{\tau^R_\text{decay} - \tau^R_\text{rise}}\left(\exp\left(-\frac{t}{\tau^R_\text{decay}}\right) - \exp\left(-\frac{t}{\tau^R_\text{rise}}\right)\right)H(t) \tag{3}$$

where $H(t)$ is the Heaviside step function, $\tau^{\text{AMPA}}_{\text{decay}} = 2$, $\tau^{\text{AMPA}}_{\text{rise}} = 0.5$, $\tau^{\text{NMDA}}_{\text{decay}} = 80$, $\tau^{\text{NMDA}}_{\text{rise}} = 2$, $\tau^{\text{GABA}}_{\text{decay}} = 5$, $\tau^{\text{GABA}}_{\text{rise}} = 0.5$, $\tau^{\text{amb}}_{\text{decay}} = 10$, and $\tau^{\text{amb}}_{\text{rise}} = 0.5$ (Nusser et al., 2001; Atallah and Scanziani, 2009; Oswald and Reyes, 2011). A random delay of mean 1 ms is imposed on $E \to E$ spikes.

0.3 Synaptic and adaptation dynamics

Here we modify the equations of section 0.2 to include synaptic and adaptation dynamics.

To model synaptic depression of I cells (Galaretta & Hestrin, 1999; Gibson et al., 1999), i.e., the phenomenon where I cells become less effective as they increase in spike rate, we vary S^{EI} as follows. If an I cell spikes at time t_0 and subsequently at time t_1, the synaptic strength of the second spike is

$$S^{EI}(t_1) = \bar{S}^{EI}\Big(1 - S^{\text{dep}} \exp\Big(-\frac{t_1 - t_0}{\tau_{\text{dep}}}\Big)\Big),$$

where $\bar{S}^{EI} = S^{EI}$ is a constant and the values of S^{dep} and τ_{dep} are given in section 0.5.

To model facilitation of layer 6 E-cells (Tarczy-Hornoch et al., 1999), the layer 6 synaptic strength S^{QL6} is computed as a function of the presynaptic cell's firing rate. When the instantaneous firing rate of a layer 6 cell at time t is r, the synaptic strength of the layer 6 cell is

$$S^{QL6}(t) = \bar{S}^{QL6} g(r), \tag{4}$$

where $\bar{S}^{QL6} = S^{QL6}$ is a constant and the function g is given in section 0.5.

To model the adaptation of E cells in layer 4 (Yuan et al., 2005), we assume the firing threshold is elevated after each spike, to be relaxed back in time to its original value. If an E neuron spikes at time t_0, then its spiking threshold follows

$$\theta_{\text{thresh}}(t) = 1 + \bar{\theta} \exp\Big(-\frac{t - t_0}{\tau_{\text{adp}}}\Big)$$

where the values of $\bar{\theta}$ and τ_{adp} are given in section 0.5.

0.4 Determination of feedback from L6 to 4Cα

Layer 6 neurons are made to spike at a rate which is modulated by the local 4Cα firing rate activity. We assume there is a response function $f(R)$ so that when R is the local layer 4 firing rate, $f(R)$ is the local layer 6 response. The function f is chosen to be sigmoidal and to fit the data at background and high contrast (Ringach et al., 2002). We stress that this response function does not depend on cortical location or on the nature of the stimulus, i.e., we assume L6 response at any location under any condition depends solely on L4 activity at that location at that point in time. The function we used in our simulations is given at the end of section 0.5.

In more detail, let $r_4(x,t)$ denote the instantaneous firing rate of a layer 4 neuron at location x and time t. We compute the local firing rate $R_4(x,t)$ by averaging over r_4 within a disc of radius 37.5 microns and over time, weighting the influence of the past by a temporal kernel $K(t)$:

$$R_4(x,t) = \frac{1}{|D|}\int_D \int_0^\infty K(s) r_4(\hat{x}, t-s)\, ds d\hat{x}, \tag{5}$$

where $|D|$ is the area of the disc, and K is a sigmoidal function in the form

$$K(t) = \frac{1}{1 + \exp\Big(\frac{t - t_{1/2}}{7}\Big)} H(t). \tag{6}$$

Here $t_{1/2}$ is a constant describing approximately how long a time-window we are averaging over. This average layer 4 rate is then used to compute

$$R_6(x,t) = f(R_4(x,t)), \tag{7}$$

where $f(R)$ is the response function above.

Finally, to simulate the effects of longer range connections in layer 6, we take a weighted average of $R_6(x,t)$ over like orientation domains: the firing rate $r_6(x,t)$ of a layer 6 cell at location x and time t is computed as

$$r_6(x,t) = \sum_{\hat{x} \in L(x)} R_6(\hat{x},t) \tag{8}$$

where $L(x)$ is a set of locations including x as well as locations in neighboring HC's with the same orientation preference. The final computed rate of the layer 6 cell at location x and time t is $r_6(x,t)$.

The implementation of this time-dependent firing rate by a L6 neuron is realized by taking a baseline spike train and copying or canceling spikes with a probability consistent with the firing rate $r_6(x,t)$. A new value $r_6(x,t)$ is recomputed every 2.5 ms.

0.5 Parameters

The parameters in Table 1 below were found by a method similar to those in Chariker et al. (2016), Methods. The remaining parameters and functions were found by studying the response properties of a small patch of neurons in the vertical preferring domain in response to vertical gratings at 4-5 different contrasts.

Table 1: Parameter values for section 0.2

Parameter	Value	Description
S^{EE}	0.023	E-to-E synaptic strength
S^{EI}	0.0476	I-to-E baseline synaptic strength
S^{IE}	0.0059	E-to-I synaptic strength
S^{II}	0.0321	I-to-I synaptic strength
S^{ELGN}	0.0506	LGN-to-E synaptic strength
S^{ILGN}	0.0805	LGN-to-I synaptic strength
S^{EL6}	0.0080	L6-to-E mean synaptic strength (failure rate up to 0.5 incorporated)
S^{IL6}	0.0020	L6-to-I synaptic strength
S^{Eamb}	0.010	ambient-to-E strength
S^{Iamb}	0.010	ambient-to-I strength
ρ^{E}_{amb}	620 kicks/sec	ambient rate to E
ρ^{I}_{amb}	500 kicks/sec	ambient rate to I

Table 2: Parameter values for synaptic and adaptation dynamics and feedback

Parameter	Value	Description
S^{dep}	0.12	Magnitude of I depression
τ_{dep}	20 ms	Relaxation time of I depression
$\bar{\theta}$	0.3170	Magnitude of E threshold adaptation
τ_{dep}	4.343	Relaxation time of E threshold adaptation
$t_{1/2}$	100 ms	Duration of temporal averaging function K defined in equation (6)

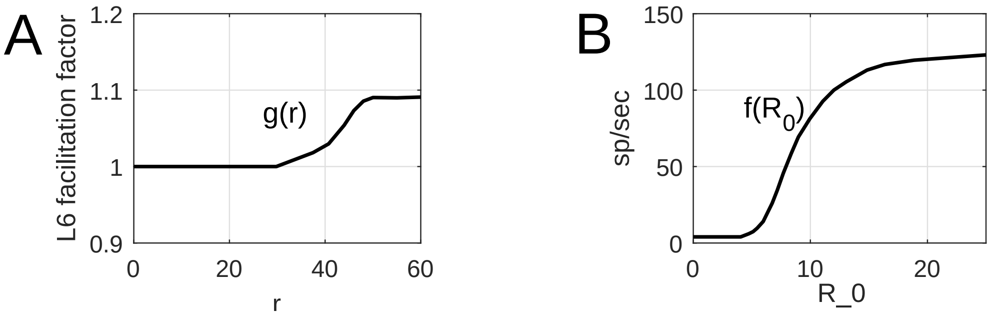

Figure 1: A) The facilitation factor as a function of firing rate for layer 6 E cells in equation (4), as discussed in section 0.3. B) The response function of layer 6 to local layer 4 activity, as introduced at the beginning of section 0.4. Here R_0 is the local firing rate of simple cells. We have elected to index layer 6 response to simple cell firing rate because they are more stable. Mean local firing rate is ≈ 1.6 times R_0.

The following references are cited in the Appendix:

References

[1] Atallah BV, Scanziani M. Instantaneous modulation of gamma oscillation frequency by balancing excitation with inhibition. Neuron. 2009;62:566–577.

[2] Chariker L, Shapley R, Young LS. Orientation Selectivity from Very Sparse LGN Inputs in a Comprehensive Model of Macaque V1 Cortex. J Neurosci. 2016;36(49):12368–12384.

[3] Enroth-Cugell C, Robson JG. The contrast sensitivity of retinal ganglion cells of the cat. J Physiol. 1966;187(3):517-552.

[4] Galaretta M, Hestrin S. A network of fast-spiking cells in the neocortex connected by electrical synapses. Nature. 1999;402:72–75.

[5] Gibson JR, Beierlein M, Connors BW. Two networks of electrically coupled inhibitory neurons in neocortex. Nature. 1999;402:75–79.

[6] Hawken MJ, Shapley R, Grosof DH. Temporal-frequency selectivity in monkey visual cortex. Vis Neurosci. 1996;13:477ÍC492.

[7] Kaplan E, Shapley R. The primate retina contains two types of ganglion cells, with high and low contrast sensitivity. Proc Nat Acad Sci USA. 1986;83(8):2755-2757.

[8] Kaplan E, Purpura K, Shapley R. Contrast affects the transmission of visual information through the mammalian lateral geniculate nucleus. J Physiol. 1987;391:267-288.

[9] Koch C. Biophysics of Computation: Information Processing in Single Neurons (Computational Neuroscience Series). New York, NY, USA: Oxford University Press, Inc.; 1999.

[10] Lin IC, Xing D, Shapley R. Integrate-and-fire vs Poisson models of LGN input to V1 cortex: noisier inputs reduce orientation selectivity. J Comput Neurosci. 2012;33(3):559-572.

[11] McLaughlin D, Shapley R, Shelley M, Wielaard DJ. A Neuronal Network Model of Sharpening and Dynamics of Orientation Tuning in an Input Layer of Macaque Primary Visual Cortex. Proc Nat Acad Sci USA. 2000;97:8087-8092.

[12] Nusser Z, Naylor D, Mody I. Synapse-specific contribution of the variation of transmitter concentration to the decay of inhibitory postsynaptic currents. Biophys J. 2001;80:1251–1261.

[13] Oswald AM, Reyes AD. Development of inhibitory timescales in auditory cortex. Cereb Cor. 2011;21:1351–1361.

[14] Ringach DL, Shapley RM, Hawken MJ. Orientation selectivity in macaque V1: diversity and laminar dependence. J Neurosci. 2002;22(13):5639–5651.

[15] Rotaru D, Yoshino H, Lweis D, Ermentrout GB, Gonzalez-Burgos G. Glutamate Receptor Subtypes Mediating Synaptic Activation of Prefrontal Cortex Neurons: Relevance for Schizophrenia. J Neurosci. 2011; 31(1):142Ď1ď76.

[16] Tarczy-Hornoch K, Martin KAC, Stratford KJ, Jack JJB. Intracortical excitation of spiny neurons in layer 4 of cat striate cortex in vitro. Cer Cortex. 1999;9:833–843.

[17] Yuan W, Burkhalter A, Nerbonne JM. Functional Role of the Fast Transient Outward K^+ Current I_A in Pyramidal Neurons in (Rat) Primary Visual Cortex. J Neurosci. 2005;25:9185–9194.

[18] Zhu W, Shelley M, Shapley R. A neuronal network model of primary visual cortex explains spatial frequency selectivity. J Comput Neurosci. 2009;26(2):217-287.
